# Inhibition of Immune Complex-Induced Inflammation by A small Molecular Weight Selectin Antagonist

**DOI:** 10.1155/S0962935194000657

**Published:** 1994

**Authors:** X. Nair, G. Todderud, L. Davern, D. Lee, A. Aruffo, K. M. Tramposch

**Affiliations:** 1 3005 First Avenue Seattle WA 98121 USA; 2 Bristol-Myers Squibb Pharmaceutical Research Institute 100 Forest Avenue Buffalo NY 14213 USA

## Abstract

The anti-inflammatory effect of a small molecular weight antagonist of P- and E-selectin-dependent cell adhesion was examined. The glycolipid sulphatide was shown to block the adherence of thrombin-activated rat platelets to HL-60 cells. This interaction is known to be dependent on P-selectin. The rat dermal reverse passive Arthus reaction was used to assess the effect of sulphatide on a neutrophil dependent inflammatory response. Sulphatide dosedependently blocked both the vascular permeability increase and cell infiltration after intraperitoneal administration. These results show that a small molecular weight compound which blocks P- and E-selectin dependent adhesion *in vitro* can effectively block the inflammation due to immune complex deposition. A compound with this type of profile may have therapeutic potential in the treatment of immune complex mediated diseases.


Mediators of Inflammation 3, 459–463 (1994)

### Inhibition of immune complex-induced inflammation by a small molecular weight selectin antagonist

X. Nair, G. Todderud, L. Davern, D. Lee, A. Aruffo[1] and K. M. Tramposch[CA]

Bristol-Myers Squibb Pharmaceutical Research Institute, 100 Forest Avenue, Buffalo, NY 14213, USA and [1]3005 First Avenue, Seattle, WA 98121, USA

[CA] Corresponding Author

**The anti-inflammatory effect of a small molecular weight antagonist of P- and E-selectin-dependent cell adhesion was examined. The glycolipid sulphatide was shown to block the adherence of thrombin-activated rat platelets to HL-60 cells. This interaction is known to be dependent on P-selectin. The rat dermal reverse passive Arthus reaction was used to assess the effect of sulphatide on a neutrophil dependent inflammatory response. Sulphatide dose-dependently blocked both the vascular permeability increase and cell infiltration after intraperitoneal administration. These results show that a small molecular weight compound which blocks P- and E-selectin dependent adhesion *in vitro* can effectively block the inflammation due to immune complex deposition. A compound with this type of profile may have therapeutic potential in the treatment of immune complex mediated diseases.**

**Key words**: Arthus reaction, Immune complexes, Selectins, Sulphatide, 3-sulphated galactosylceramide

### Introduction

During an inflammatory response leukocytes must adhere to the vascular endothelium before migrating into the extravascular space. P- and E-selectin are cell surface receptors that are expressed on activated endothelial cells.[1,2] P-selectin is also expressed on activated platelets.[1] The selectins mediate, in part, the initial adhesion of leukocytes to the endothelial wall.[3] These receptors have a similar extracellular domain structure including an N-terminal lectin-like domain, an epidermal growth factor related domain and several complement regulatory protein repeat elements.[4,5] The lectin domain is analogous to other C-type $Ca^{2+}$-dependent lectins, suggesting that cell–cell adhesion mediated by selectins is likely to involve carbohydrate:protein interactions. P- and E-selectin are known to recognize sialylated, fucosylated lactosaminoglycans such as sialyl Lewis x ($sLe^x$).[6–8] Therefore, carbohydrate based ligands are a reasonable starting point in efforts to identify antagonists of selectin-mediated adhesion. Such antagonists could be efficient inhibitors of leukocyte extravasation at sites of inflammation.

A recombinant P-selectin IgG chimera has been shown to bind to the glycolipid sulphatide.[4] The finding that sulphatide can block P-selectin dependent adhesion of U937 cells[4] and human PMNs,[9] suggested that this glycolipid could have anti-inflammatory activity. We now report that sulphatide effectively inhibits the increase in vascular permeability and cell infiltration in the dermal reverse passive Arthus reaction in rats. Sulphatide exemplifies a simple class of carbohydrate-containing compounds which appear to act via this new anti-inflammatory strategy.

### Materials and Methods

*Test compounds*: Sulphatide (Bovine Brain) was purchased from Sigma Chemical and was supplied as a mixture of 3-sulphated galactosylceramides. The N-acyl fatty acid was primarily nervonic acid (70%) and the minor component contained lignoceric acid (30%). Sulphatide was tested for endotoxin contamination using a *Limulus* amoebocyte lysate assay. The endotoxin contamination was 19 EU/5 mg sulphatide. Lipopolysaccharide from *Escherichia coli* serotype 0127:B8 and galactosylceramide were purchased from Sigma.

*Animals*: Male Sprague–Dawley specific pathogen free rats (250–300 g) were obtained from Hill Top Lab Animals (Scottdale, PA). The rats were housed individually in stainless steel cages and in accordance with NIH guidelines. The animals were given free access to food and water.

*The effect of sulphatide on HL-60 cell binding to immobilized selectin-Ig*: The construction of soluble fusion proteins of the extracellular domain of P- and E-selectins has been described.[4,10] These proteins were purified from the medium of transfected cos cells. The proteins contain the human lectin domain, the EGF domain, and two complement repeats of the human selectins fused to the hinge, CH1 and CH2 domains of human IgG1. The assay for cell binding to immobilized selectin receptor globulin was per-

© 1994 Rapid Communications of Oxford Ltd formed as described.[9] Briefly, the wells of a 96-well dish (Corning) were coated overnight with anti-human Fc antibody diluted into 50 mM Tris pH 9.1 buffer, blocked with 1% non-fat dry milk in Dulbecco's phosphate buffered saline (DPBS), and allowed to bind selectin Rg. Cells were labelled with 10 μM calcein acetoxymethyl ester (Molecular Probes) for 30 min at $3 \times 10^7$ cells per ml at room temperature. The blocked Rg-bound wells were rinsed twice, and labelled cells were added for 30 min at room temperature. Unbound cells were removed by aspiration and three washes of the wells. Fluorescence in each well was determined using a Millipore Cytofluor fluorescent plate reader. Sulphatide (Sigma) was prepared as a 20 mg/ml stock solution in dimethyl sulphoxide (DMSO), diluted in DPBS to 2 mg/ml working solution, and briefly sonicated prior to use. When sulphatide or other inhibitors were tested, the selectin-Ig coated wells were preincubated at room temperature for 15 min with the inhibitor, and 200 000 cells were added, to yield the final indicated inhibitor concentration in 160 μl of DPBS. Sulphatide had no effect on the amount of selectin-Ig bound to the plate (G. Todderud, unpublished observation).

*Effect of sulphatide on the binding of activated rat platelets to HL-60 cells*: Male Sprague–Dawley rats (250–300 g) were anaesthetized with metofane (Pitman-Moore). The animals were exsanguinated by drawing blood from the aorta into EDTA-containing vacutainer tubes. Platelet-rich plasma was prepared by centrifugation at 120 × **g** for 20 min. The platelets were isolated by centrifuging the plasma at 1 200 × **g** for 20 min. The platelet pellet was resuspended in THBE buffer (Tyrodes salts, 5 mM HEPES, 10 mM EDTA, and 0.2% BSA). The isolated platelets were fluorescently labelled by incubation with 10 μM calcein acetoxymethyl ester (Molecular Probes) at 37°C for 15 min. The labelled platelets were pelleted and resuspended in THB (same buffer as THBE except without EDTA) and activated with thrombin (2 units/ml, at $10^7$ cells/ml) for 10 min at 37°C. HL-60 cells and platelets were fixed in 1% formalin for 30 min. The HL-60 cells were then added to the platelets and incubated at a ratio of 5 platelets per HL-60 in the presence or absence of the indicated concentration of inhibitor for 30 min. The adhesion was determined on a FACScan flow cytometer, by analysing the forward scatter and fluorescent intensity of the mixed population. The platelets, which appear at lower forward scatter values, were excluded by gating only the higher forward scatter HL-60 cell present. The HL-60 cells with high fluorescent intensity contain bound platelets. The percentage of HL-60 cells in fluorescent and non-fluorescent populations was determined, and plotted as a function of inhibitor concentration.

*The effect of sulphatide on the rat dermal reverse passive Arthus reaction*: Male Sprague–Dawley rats with jugular vein cannulae (250–300 g) were anaesthetized with a mixture of 100 mg/kg Ketaset (Fort Dodge Laboratories) and 4 mg/kg Rompun (Miles, Inc.) given intraperitoneally. Closely clipped dorsal skin was injected intradermally (i.d.) with 0.6 mg Anti-BSA (Sigma). Negative control animals received an intradermal injection of saline. The antigen, BSA (10 mg) was then administered via the jugular vein. The BSA contained 1 μCi of $^{125}$I-BSA (specific activity = 1–5 μCi/μg). Sulphatide, galacto-sylceramide or vehicle (0.5% Tween 80 in normal saline) was administered by intraperitoneal injection 2 h prior to intravenous BSA administration. The rats were killed at 4 h by $CO_2$ inhalation and 15 mm punch biopsies of the reaction sites were taken. To assess changes in vascular permeability the $^{125}$I content of the biopsies was determined by gamma scintillation spectroscopy. Permeability indices were calculated by measuring the ratio of radioactivity in full thickness skin biopsies compared with radioactivity present in 1 μl of plasma. The skin accumulation of neutrophils was determined from the tissue myeloperoxidase (MPO) content. The biopsy MPO content was determined using a modified version of the method described by Bradley *et al.*[11] Each 15 mm punch biopsy was homogenized with a Brinkman Polytron homogenizer in 10 ml of 0.5% hexadecyltrimethyl ammonium bromide (HTAB) in 0.05 M potassium phosphate buffer (pH 6.0). After a single freeze/thaw step the homogenate was sonicated for 20 s and centrifuged at 1 000 **g** for 10 min. A 0.05 ml aliquot of the supernatant was assayed by mixing with 0.15 ml O-dianisidine (0.334 mg/ml) and 0.0005% hydrogen peroxide in 0.05 M potassium phosphate buffer. Change in absorbance at 450 nm was measured at room temperature using a $V_{max}$ kinetic plate reader (Molecular Devices, Palo Alto, CA). The results are expressed as the mean mOD/min/biopsy. Selected skin tissues were evaluated histologically. Skin biopsies were fixed in 10% neutral buffered formalin, processed in the usual way, embedded in paraffin, cut into 4 μm thick sections, then stained with haematoxylin and eosin.

### Results and Discussion

Sulphatide is a mixture of 3-sulphated galactosyl ceramides in which the N-acyl fatty acid is primarily nervonic and lignoceric acid (Fig. 1). Previous studies have demonstrated the importance of the sulphate group in binding P-selectin and inhibiting P-selectin dependent adhesion *in vitro*. We therefore used galactosylceramide as a negative control in the *in vivo* experiments. Figure 2 demonstrates that sulphatide can block the binding of HL-60 cells to plates coated with soluble P- or E-selectin Ig fusion

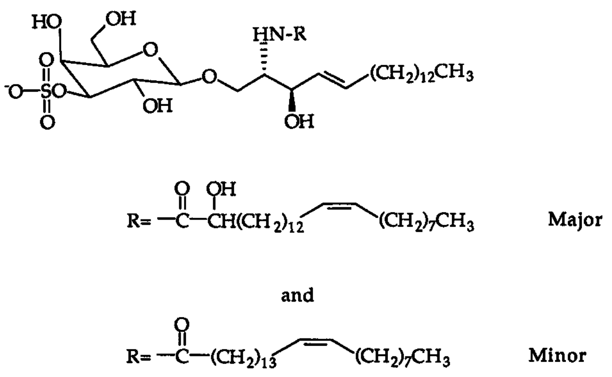

FIG. 1. Structure of sulphatide.

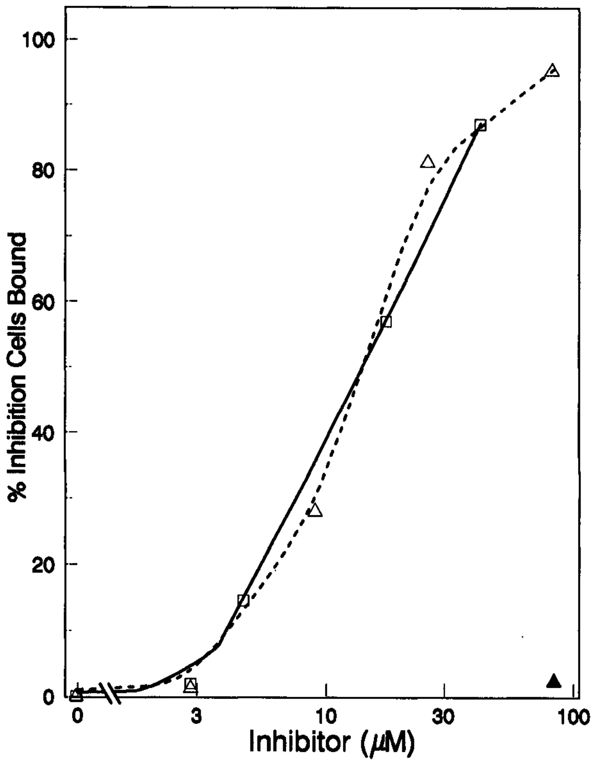

FIG. 2. Inhibition of binding of HL-60 cells to immobilized P-selectin-Ig (□) or E-selectin-Ig (△) by sulphatide compared with binding of P-selectin-Ig in the presence of galactosylceramide (▲). The number of fluorescent labelled HL-60 cells bound to the plate coated with selecting-Ig was determined in triplicate at each concentration tested. The standard deviations are within the symbols, and this is representative of four separate determinations.

proteins. Galactosylceramide had no effect. The demonstration that sulphatide can block E-selectin-dependent adhesion is interesting in view of the reported finding that E-selectin does not bind to immobilized sulphatide.[4,6] However, it is known that sulphated analogues of sLex can bind to E-selectin with a greater affinity than sLex.[12] Platelets are known to bind to HL-60 cells in a P-selectin dependent manner.[13] To show that sulphatide can block a cell–cell interaction, we evaluated the effect on the adhesion of activated rat platelets to HL-60 cells by flow cytometry. Figure 3 shows that sulphatide dose-dependently blocks this cell–cell adhesion. Taken

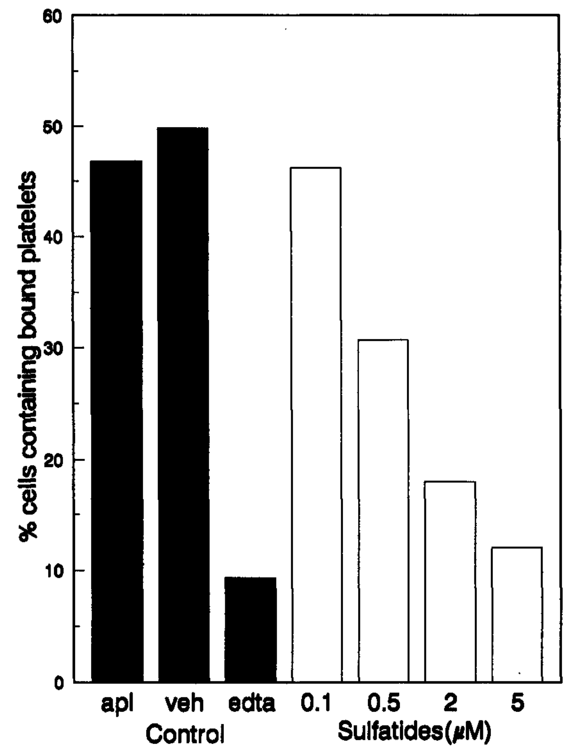

FIG. 3. Inhibition of binding of activated rat platelets to HL-60 cells. The percentage of HL-60 cells which contain bound activated platelets (apl) was determined by flow cytometry. The closed bars show the result of treatment with the DMSO vehicle (veh) or 10 mM EDTA (edta), and binding in the presence of the indicated concentrations of sulphatide is shown by the open bars.

together, these data show that sulphatide inhibits both human P- and E-selectin and that the effect can be extended across species to rat P-selectin.

The reverse passive Arthus reaction is an immediate hypersensitivity reaction initiated by immune complex depositions in the vascular wall followed by complement fixation resulting in increased vascular permeability and polymorphonuclear leukocyte (PMN) infiltration.[14] This acute reaction may be involved in autoimmune diseases characterized by immune complex deposition. For example, in rheumatoid arthritis circulating and intraarticular immune complexes appear to play a role in the pathogenesis of the disease.[15] E-selectin has been shown to be involved in the inflammation resulting from an Arthus reaction in both rat skin and lungs.[16] The role of P-selectin in the Arthus reaction has not been determined. However, P-selectin does play a role in neutrophil dependent lung injury,[17] and P-selectin deficient mice exhibit delayed inflammatory reactions[18] suggesting a role for this adhesion molecule in inflammation. Table 1 shows the dose-related inhibitory effect of sulphatide on vascular permeability and PMN infiltration. By contrast, the nonsulphated galactosylceramide at 100 mg/kg had no effect on vascular permeability or PMN infiltration. In data not shown, sulphatide had no effect on circulating PMN numbers. In addition, the anti-inflammatory effect was not due to endotoxin contami-

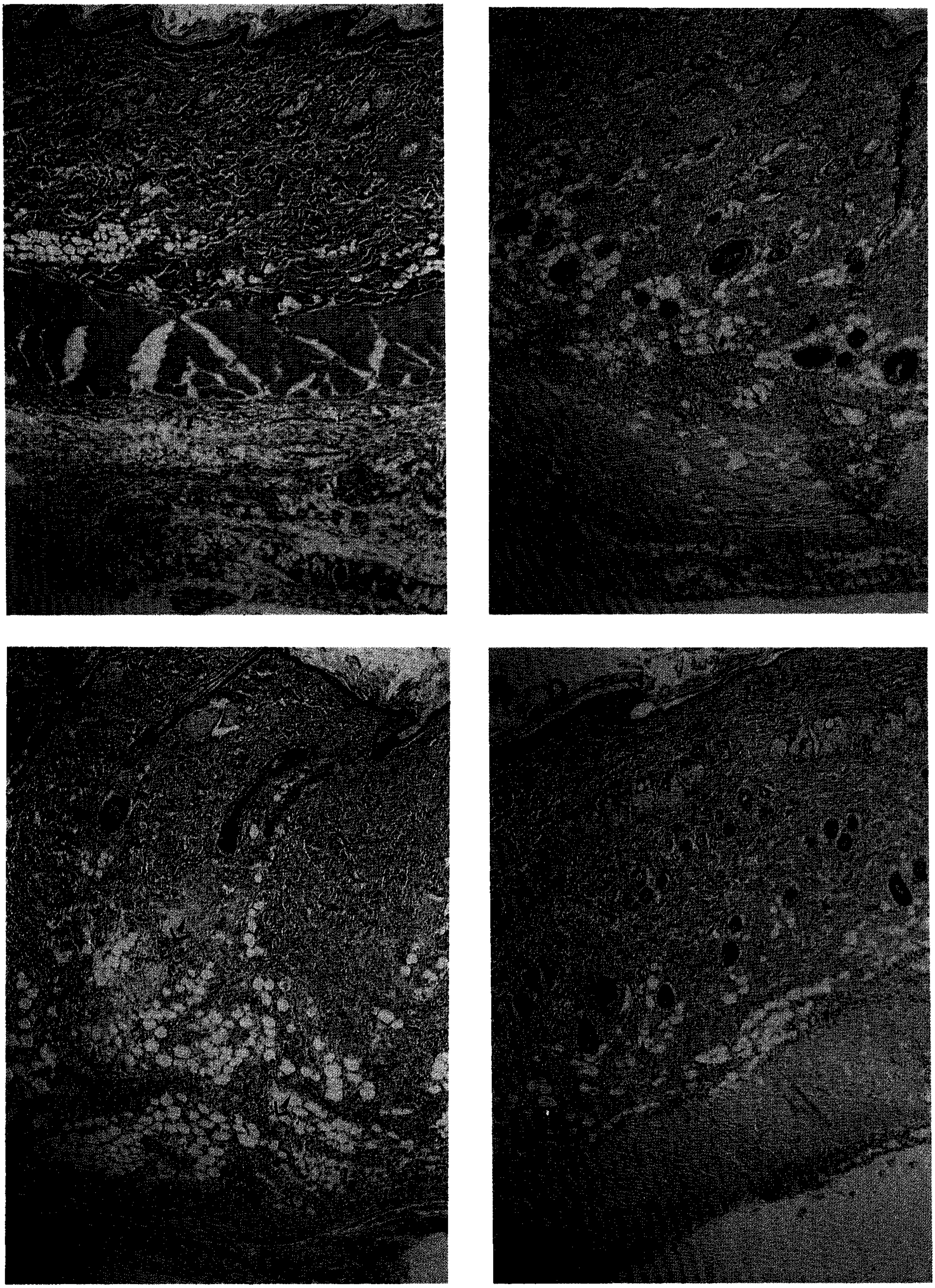

FIG. 4. Light micrographs of H&E stained rat skin following Arthus reaction (magnification x 90). Compared with the negative control (A) the intradermal sites in animals treated with the vehicle (B) and galactosylceramide (C) showed cell infiltration as indicated by the arrows. Compared with the vehicle treated group, animals treated with sulphatide (D) showed a reduction in infiltrating cells. Rats were administered sulphatide (100 mg/kg), galactosylceramide (100 mg/kg) or vehicle as described in the legend to Table 1. Full thickness skin biopsies fixed in 10% buffered formalin were prepared as vertical sections and stained with H&E.

**Table 1**. The effect of sulphatide and galactosylceramide on the rat dermal reverse passive Arthus reaction

| | Dose (mg/kg)[a] | Vascular permeability[b] | PMC[c] infiltration |
|---|---|---|---|
| Vehicle control | – | 287 ± 8 | 363 ± 46 |
| Sulphatide | 5 | 278 ± 26 | 330 ± 48 |
| | 20 | 294 ± 16 | 194 ± 36* |
| | 50 | 266 ± 13 | 179 ± 36* |
| | 100 | 231 ± 19 | 145 ± 25* |
| Galactosylceramide | 100 | 286 ± 8 | 404 ± 50 |

[a]Test materials were administered by intraperitoneal injection 2 h prior to anti-BSA injection. The vehicle was composed of 0.5% Tween 80 in normal saline.
[b]Vascular permeability increases were measured as the accumulation of tissue $^{125}$I-BSA. The values represent mean µl of plasma ± SEM ($n$ = 8).
[c]Relative PMN infiltration was quantitated from the tissue myeloperoxidase (MPO) level. The values represent mean MPO activity (mOD/min/biopsy) ± SEM ($n$ = 8).
*$p$ > 0.05 compared with vehicle control. Statistical differences were analysed by paired Student's $t$-test.

nation since administration of LPS in doses of at least 96 EU/kg had no effect on the vascular permeability or cell infiltration parameters (X. Nair, personal observation). Figure 4 shows the microscopic changes induced by the RPA reaction in rat skin. Extensive extravascular cell infiltration and dermal oedema are apparent in the reaction sites following vehicle or galactosylceramide administration. In contrast, sulphatide caused a marked reduction in cell infiltration compared with vehicle and galactosylceramide.

These results show that a small molecular weight compound which blocks P- and E-selectin dependent cell adhesion *in vitro* can effectively block the inflammation due to immune complex deposition. L-selectin has also been shown to bind sulphatide.[6,10] Inhibition of L-selectin-dependent adhesion by sulphatide could contribute to the anti-inflammatory effect since soluble L-selectin chimeras and anti-L-selectin F(ab′)$_2$ antibody fragments have been shown to block PMN infiltration in several animal models.[19,20] Therefore, the results we report here do not shed additional light on the relative roles of P-, E- and L-selectin in PMN recruitment. However, these observations support the concept that compounds which block selectin dependent adhesion may have therapeutic potential in the treatment of acute inflammatory reactions where PMN infiltration is predominant.

### References

1. McEver RP. Leukocyte interactions mediated by selectins. *Thromb Haemost* 1991; **66**: 80–87.
2. Lasky LA. Selectins: interpreters of cell-specific carbohydrate information during inflammation. *Science* 1992; **258**: 964–969.
3. Bevilacqua MP, Nelson RM. Selectins. *J Clin Invest* 1993; **91**: 379–387.
4. Aruffo A, Kolanus W, Walz G, Fredman P, Seed B. CD62/P-selectin recognition of myeloid and tumor cell sulfatide. *Cell* 1991; **67**: 35–44.
5. Johnson GI, Cook RG, McEver RP. Cloning of GMP-140, a granule membrane protein of platelets and endothelium: sequence similarity to proteins involved in cell adhesion and inflammation. *Cell* 1989; **56**: 1033–1044.
6. Foxall C, Watson SR, Dowbenko D, Fennie C, Lasky LA, Kiso M, Hasegawa A, Asa D, Brandley BK. The three members of the selectin receptor family recognize a common carbohydrate epitope, the sialyl Lewis (x) oligosaccharide. *J Cell Biol* 1992; **117**: 895–902.
7. Phillips ML, Nudelman E, Gaeta FCA, Perez M, Singhai AK, Hakomari S, Paulson JC. ELAM-1 mediates cell adhesion by recognition of a carbohydrate ligand, Sialyl-Le$^x$. *Science* 1990; **250**: 1130–1132.
8. Polley MJ, Phillips ML, Wayner E, Nudelman E, Singhal K, Hakomari SI, Paulson JC. CD62 and endothelilal cell-leukocyte adhesion molecule 1 (ELAM-1) recognize the same carbohydrate ligand, sialyl-Lewis$^x$. *Proc Natl Acad Sci USA* 1991; **88**: 6224–6228.
9. Todderud G, Alford J, Millsap KA, Aruffo A, Tramposch KM. PMN binding to P-selectin is inhibited by sulfatide. *J Leuk Biol* 1992; **52**: 85–88.
10. Walz G, Aruffo A, Kolanus W, Bevilacqua M, Seed B. Recognition by ELAM-1 of the Sialyl-Le$^x$ determinant on myeloid and tumor cells. *Science* 1990; **250**: 1132–1135.
11. Bradley PP, Priebat DA, Christensen RD, Rothstein G. Measurement of cutaneous inflammation: estimation of neutrophil content with an enzyme marker. *J Invest Dermatol* 1982; **78**: 206–209.
12. Yuen CT, Lawson AM, Chai WL, Larkin M, Stoll MJ, Stuart AC, Sullivan FX, Ahern TJ, Feizi T. Novel sulfated ligands for the cell adhesion molecule E-selectin revealed by the neoglycolipid technology among O-lined oligosaccharides on an ovarian cystandenoma glycoprotein. *Biochemistry* 1992; **31**: 9126–9131.
13. Larsen E, Celi A, Gilbert GE, Furie BC, Erban JK, Bonfanti R, Wagner DD, Furie B. PADGEM protein: a receptor that mediates the interaction of activated platelets with neurophils and monocytes. *Cell* 1989; **59**: 305–312.
14. Cochrane CG, Aikin BS. Polymorphonuclear leukocytes in immunologic reactions. The destruction of vascular basement membrane *in vivo* and *in vitro*. *J Exp Med* 1966; **124**: 733–752.
15. Zvaifler NJ. The immunopathology of joint inflammation in rheumatoid arthritis. *Adv Immun* 1973; **16**: 265–336.
16. Mulligan MS, Varani J, Dame MK, Lane CL, Smith CW, Anderson DC, Ward PA. Role of endothelial-leukocyte adhesion molecule 1 (ELAM-1) in neutrophil-mediated lung injury in rats. *J Clin Invest* 1991; **88**: 1396–1406.
17. Mulligan MS, Polley MJ, Bayer RJ, Nunn MF, Paulson JC, Ward PA. Neutrophil-dependent acute lung injury. Requirement for P-selectin (GMP-140). *J Clin Invest* 1992; **90**: 1600–1607.
18. Mayadas TN, Johnson RC, Rayburn H, Hynes RO, Wagner DD. Leukocyte rolling and extravasation are severely compromised in P-selectin-deficient mice. *Cell* 1993; **74**: 541–554.
19. Watson SR, Fennie C, Lasky LA. Neutrophil influx into an inflammatory site inhibited by a soluble homing receptor-IgG chimera. *Nature* 1991; **349**: 164-167.
20. Mulligan MS, Miyasaki M, Tamatani T, Jones ML, Ward PA. Requirements for L-selectin in neutrophil-mediated lung injury in rats. *J Immunol* 1994; **152**: 832–840.

**Received 8 July 1994;**
**accepted in revised form 30 August 1994**
